# Simulation‐based team‐training in acute stroke: Is it safe to speed up?

**DOI:** 10.1002/brb3.2814

**Published:** 2022-11-23

**Authors:** Liv Jorunn Høllesli, Soffien Chadli Ajmi, Martin W. Kurz, Thomas Bailey Tysland, Morten Hagir, Ingvild Dalen, Sigrun Anna Qvindesland, Hege Ersdal, Kathinka D. Kurz

**Affiliations:** ^1^ Stavanger Medical Imaging Laboratory (SMIL) Department of Radiology Stavanger University Hospital Stavanger Norway; ^2^ Department of Electrical Engineering and Computer Science University of Stavanger Stavanger Norway; ^3^ Neurology Research Group, Department of Neurology Stavanger University Hospital Stavanger Norway; ^4^ Department of Quality and Health Technology University of Stavanger Stavanger Norway; ^5^ Department of Clinical Medicine University of Bergen Bergen Norway; ^6^ Department of Radiology Hospital of Southern Norway Kristiansand Kristiansand Norway; ^7^ Department of Research Section of Biostatistics Stavanger University Hospital Stavanger Norway; ^8^ Department of Research, Simulation Section Stavanger University Hospital Stavanger Norway; ^9^ Critical Care and Anesthesiology Research Group Stavanger University Hospital Stavanger Norway; ^10^ Faculty of Health Sciences University of Stavanger Stavanger Norway

**Keywords:** intracranial hemorrhages, ischemic stroke, patient safety, simulation training, thrombolytic therapy

## Abstract

**Background:**

In acute ischemic stroke (AIS), rapid treatment with intravenous thrombolysis (IVT) is crucial for good clinical outcome. Weekly simulation‐based team‐training of the stroke treatment team was implemented, resulting in faster treatment times. The aim of this study was to assess whether this time reduction led to a higher proportion of stroke mimics (SMs) among patients who received IVT for presumed AIS, and whether these SM patients were harmed by intracranial hemorrhage (ICH).

**Methods:**

All suspected AIS patients treated with IVT between January 1, 2015 and December 31, 2020 were prospectively registered. In 2017, weekly in situ simulation‐based team‐training involving the whole stroke treatment team was introduced. To analyze possible unintended effects of simulation training, the proportion of SMs among patients who received IVT for presumed AIS were identified by clinical and radiological evaluation. Additionally, we identified the extent of symptomatic ICH (sICH) in IVT‐treated SM patients.

**Results:**

From 2015 to 2020, 959 patients were treated with IVT for symptoms of AIS. After introduction of simulation training, the proportion of patients treated with IVT who were later diagnosed as SMs increased significantly (15.9% vs. 24.4%, *p* = .003). There were no ICH complications in the SM patients treated before, whereas two SM patients suffered from asymptomatic ICH after introduction of simulation training (*p* = 1.0). When subgrouping SMs into prespecified categories, only the group diagnosed with peripheral vertigo increased significantly (2.5% vs. 8.6%, *p* < .001).

**Conclusions:**

Simulation training of the acute stroke treatment team was associated with an increase in the proportion of patients treated with IVT for a suspected AIS who were later diagnosed with peripheral vertigo. The proportion of other SM groups among IVT‐treated patients did not change significantly. No sICH was detected in IVT‐treated SM patients.

## 1 INTRODUCTION

Ischemic stroke is a major cause of morbidity and mortality worldwide (Feigin et al., 2017; Wafa et al., 2020). The global burden of stroke is increasing, especially in younger age groups in low–moderate‐income countries (Katan & Luft, 2018; Virani et al., 2020). The main therapeutic target in acute ischemic stroke (AIS) is timely recanalization of the occluded vessel. Rapid recognition of stroke symptoms, patient transfer, diagnostics, and acute treatment, including intravenous thrombolysis (IVT) and/or endovascular thrombectomy (EVT), are of vital importance and significantly improve outcomes in stroke patients (Powers et al., 2019). The therapy should be given as fast as possible, as the clinical outcome improves if therapy is given timely (Advani et al., 2017; Meretoja et al., 2014, 2017). In order to reduce treatment times in AIS patients, we have introduced weekly in situ simulation‐based team‐training sessions for the stroke treatment team. This led to a reduction of the median door‐to‐needle time (DNT) from 27 to 13 minutes and reduced patient morbidity and mortality (Ajmi et al., 2019).

Stroke mimics (SMs) are common and comprise up to 43% of patients presenting with acute neurological symptoms in the prehospital phase (McClelland et al., 2019; Tarnutzer et al., 2017). Due to the possibility of bleeding complications, IVT treatment of SMs poses a potential risk for the patients (Hacke et al., 2008; National Institute of Neurological Disorders and Stroke rt‐PA Stroke Study Group, 1995); furthermore, the treatment is associated with an increased resource and cost burden to hospitals and society (Goyal et al., 2015).

The aim of this study was to evaluate whether simulation training for the stroke treatment team led to a higher proportion of SMs among patients who received IVT for presumed AIS, and whether these patients were harmed by intracranial hemorrhage (ICH).

## 2 MATERIALS AND METHODS

### 2.1 Patients

All patients with suspected AIS treated with IVT between January 1, 2015 and December 31, 2020 were prospectively registered in a local research database. All relevant data‐endpoints, including ICH and the final diagnosis, were registered in this database. On February 1, 2017, repeated clusters with weekly in situ simulation‐based team‐training involving the stroke treatment team were introduced. Patients treated before the introduction of the simulation training served as control group.

Vertigo patients were IVT treated if the symptoms were deemed to be of cerebrovascular origin. The IVT decision was made by the neurologic team on call (resident and senior consultant) and comprised both clinical findings and results of the radiological investigations.

### 2.2 Simulation training

We performed clusters of in situ simulation‐based team‐training using acute stroke scenarios, simulating both the emergency medical services (EMS) and the in‐hospital stroke care pathway from calling the EMS to administration of IVT. The simulated cases presented with FAST symptoms (face–arm–speech, and aphasia or dysarthria). Vertigo symptoms were not simulated. All in‐hospital stroke treatment team members and paramedics on‐call participated in the simulation training. Our primary aims were to reduce treatment times, to enhance protocol adherence, and to improve communication skills. Details of the simulation training have been described previously (Ajmi et al., 2019).

### 2.3 Radiological evaluation

Patients with suspected AIS were routinely investigated with a CT protocol comprising non‐contrast CT of the head, CT angiography of the precerebral and intracranial arteries and CT perfusion immediately after hospital admission. In most cases, this is followed by magnetic resonance imaging (MRI), including diffusion‐weighted imaging (DWI) during the first days after hospital admission. Supplementary CT or MRI examinations were performed in patients with clinical deterioration. In most patients with suspected wake‐up stroke or with unknown symptom onset time, MRI, including DWI and fluid‐attenuated inversion recovery, was routinely used as a first‐line diagnostic tool. If the neurological symptoms were deemed to be of cerebrovascular origin, patients were IVT treated also in cases of negative initial MRI (Edlow et al., 2017).

### 2.4 Clinical evaluation

Neurological impairment on admission was assessed using the National Institutes of Health Stroke Scale (NIHSS). NIHSS was performed at hospital admission, repeatedly after IVT, and on the day of hospital discharge. Functional outcome at 3 months was evaluated using modified Rankin Scale (mRS) assessed by a certified stroke nurse by means of a telephone interview. Good functional outcome was defined as mRS score 0–2, and poor functional outcome was defined as mRS score 3–6.

SMs were defined as patients who presented with stroke‐like symptoms, but after diagnostic work‐up were determined not to have suffered from a stroke episode. Evaluation of the clinical course, examination by all specialists involved (typically neurologists, ear, nose and throat specialists, or cardiologists), and evaluation with MRI were all part of the diagnostic work‐up. SMs were diagnosed during their hospital stay and then retrospectively categorized into eight different subgroups: psychiatric disorders, peripheral vertigo, epilepsy, migraine, infectious diseases, intoxication, peripheral facial palsy and other.

ICH was classified according to the European Cooperative Acute Stroke Study II (ECASS II) (Hacke et al., 1998); symptomatic ICH (sICH) was defined as ICH associated with a NIHSS deterioration of four points or more.

### 2.5 Statistics

All statistical analyses were performed using SPSS Statistics version 24 (IBM Cooperation, Armonk, NY, USA). Categorical variables are presented as count (percent, %) and continuous variables as median (interquartile range, IQR). Changes in proportions were evaluated using Pearson Chi‐squared test or Fisher's exact test, as appropriate. Changes in continuous variables were evaluated using the Mann–Whitney test.

### 2.6 Ethical approval

All procedures involving human participants were performed in accordance with the ethical standards of the institutional and/or national research committee and with the 1964 Helsinki Declaration and its later amendments or comparable ethical standards. This study was approved by the regional ethical committee and the local hospital authorities. Informed consent was waived, after approval of the regional ethical committee.

## 3 RESULTS

From 2015 to 2020, 959 patients were treated with IVT under suspicion of AIS (Table 1). The proportion of IVT treatments varied between 22.8% and 30.8%.

**TABLE 1:** Number of stroke admissions, intravenous thrombolysis (%) and stroke mimics (%) between 2015 and 2020

	2015	2016	2017	2018	2019	2020
Stroke admissions	438	578	674	614	498	627
IVT treatments (%)	127 (29.0)	178 (30.8)	182 (27.0)	140 (22.8)	140 (28.1)	192 (30.6)
Stroke mimics (%)	16 (12.6)	33 (18.5)	27 (14.8)	40 (28.6)	45 (32.1)	46 (24.0)
DNT, median (IQR)	30 (22, 45) (*n* = 113)	25 (17, 37) (*n* = 175)	15 (9, 29) (*n* = 174)	17 (11, 30) (*n* = 136)	16 (11, 30) (*n* = 137)	20 (13, 35) (*n* = 184)

*Note*: Stroke mimics: Percent is calculated from the number of patients IVT treated for suspected acute ischemic stroke. Simulation‐based team‐training started on February 1, 2017.

Abbreviations: DNT, door‐to‐needle time (all IVT‐treated patients, including wake‐up and in‐house stroke); IQR, interquartile range; IVT, intravenous thrombolysis.

In 2015 and 2016, the years before introduction of simulation‐based team‐training, the proportion of SMs among IVT‐treated patients was 12.6% and 18.5%, respectively. In 2017, the year simulation training started, the proportion of SMs was 14.8%. Between 2018 and 2020, years with ongoing simulation training, the proportion of SMs was 27.8% (24.0%–32.1%, Table 1). A significant increase was registered between 2017 and 2018 (*p* = .003). 2018 was the first complete year with simulation training. Treatment times dropped significantly between 2016 and 2017, after introduction of the simulation training (*p* < .001) (Table 1).

Baseline characteristics and cerebrovascular risk factors of patients treated with IVT are described in Table 2. Before introduction of simulation‐based team‐training in February 2017, 320 patients were treated with IVT, 639 patients thereafter (Table 1). Patients treated with IVT after introduction of simulation‐based team‐training had significantly less atrial fibrillation (*p* = .004); otherwise, there were no statistically significant differences in baseline variables (Table 2).

**TABLE 2:** Patient baseline characteristics and cerebrovascular risk factors before and after implementation of simulation training

	Before simulation training (*n* = 320)	After simulation training (*n* = 639)	*p*‐Value
Age, median (IQR)	71 (59, 81)	69 (55, 80)	.076
Sex—female	135 (42.2)	296 (46.3)	.23
Hypertension	145 (45.3)	259 (40.5)	.16
Diabetes	40 (12.5)	89 (13.9)	.54
Atrial fibrillation	47 (14.7)	55 (8.6)	.004
Prior stroke	71 (22.2)	132 (20.7)	.58

*Note*: Data presented as count (%) unless otherwise stated.

Abbreviation: IQR, interquartile range.

The clinical outcome of the patients before and after implementation of simulation‐based team‐training did not differ in our patient group (Table 3). The proportion of SMs among IVT‐treated patients rose from 51 (15.9%) before introduction of simulation‐based team‐training to 156 (24.4%) thereafter (*p* = .003). There were no bleeding complications in the SM patients treated before introduction of simulation‐based team‐training, whereas two (1.3% of 156) SM patients suffered from asymptomatic ICH after introduction of simulation training (*p* = 1.0). The ICH in one patient had a diameter of 5 mm and was located in the right frontal lobe, whereas the other patient was diagnosed with a small amount of blood in the subarachnoid space parieto‐occipitally on the right side. None of the hemorrhages were accompanied with neurological deterioration. The two SM patients with ICH had a DNT of 22 and 49 min, respectively.

**TABLE 3:** Clinical outcome before and after implementation of simulation training

	Before simulation training (*n* = 320)	After simulation training (*n* = 639)	*p*‐Value
NIHSS score at admission. Median, (IQR)	3 (2, 7) (*n* = 313)	3 (1, 6) (*n* = 627)	.023
NIHSS score at dismissal. Median, (IQR)	0 (0, 1) (*n* = 279)	0 (0, 1) (*n* = 579)	.50
mRS score 0–2 at 3 months. *N* (%)	179 (72.8) (*n* = 246)	321 (75.2) (*n* = 428)	.49

Abbreviations: IQR, interquartile range; mRS, modified Rankin Scale; *N* = number of patients; NIHSS, National Institutes of Health Stroke Scale.

When subgrouping SMs into prespecified categories, there was a significantly higher proportion of patients with peripheral vertigo (8 patients/2.5% vs. 55 patients/8.6%, *p* < .001) among IVT‐treated patients (Table 4) after implementation of simulation training. There were no significant changes in the proportion of other SM categories.

**TABLE 4:** Intravenous thrombolysis (IVT)‐treated stroke mimic patients and final diagnoses before and after implementation of simulation training

	Before simulation training (*n* = 320)	After simulation training (*n* = 639)	*p*‐Value
Stroke mimics total	51 (15.9)	156 (24.4)	.003
Psychiatric disorders	8 (2.5)	18 (2.3)	.88
Peripheral vertigo	8 (2.5)	55 (8.6)	<.001
Epilepsy	3 (0.9)	15 (2.3)	.13
Migraine	9 (2.8)	23 (3.6)	.69
Infectious diseases	3 (0.9)	6 (0.9)	1.00
Intoxication	2 (0.6)	3 (0.5)	1.00*
Peripheral facial palsy	2 (0.6)	3 (0.5)	1.00*
Other	16 (5.0)	33 (5.2)	.54

*Note*: Data presented as count (%). *p*‐Values from Chi‐squared test, except * Fisher's exact test.

## 4 DISCUSSION

Introduction of simulation‐based team‐training in 2017 was accompanied by a significant increase in the proportion of patients treated with IVT for a presumed AIS who were later diagnosed as SMs (15.9% vs. 24.4%, *p* = .003), consisting mainly of patients with peripheral vertigo. This increase was not seen during the first year of simulation training (Ajmi et al., 2019). Possibly, the rise has been supported by an evolving fear to harm patients by withholding IVT in ambiguous cases, by some insecurity in the clinical evaluation of vertigo patients, and by the recognition that IVT in SMs is rarely associated with complications (Moulin & Leys, 2019).

The overall number of stroke admissions showed a tendency to increase during the period 2015–2020, with an exception of 2019, whereas the proportion of IVT treatments fluctuated. The increasing number of stroke admissions may be explained by a general increased awareness of stroke symptoms and the importance of rapid treatment. We do not have a clear explanation for the decrease in the number of stroke admissions in 2019, nor the fluctuation in the proportion of IVT treatments.

Before the start of simulation‐based team‐training, SM rates at our center were in the range of centers with CT‐based patient selection (Kvistad et al., 2019). Although the numbers increase thereafter, they are still comparable to other centers (Pohl et al., 2021; Sporns et al., 2021). It is well known that the proportion of SMs among IVT‐treated patients under the suspicion of AIS varies considerably and also depends on the initial imaging modality used. Centers with initial CT examination, and centers actively reducing treatment times, in general have higher SM rates ranging up to 30% (Burton et al., 2017; Liberman et al., 2015). The increase of SM rates is likely to be multifactorial and related to a focus on time, the acceptance of lower diagnostic specificity, and an increased comfort to perform IVT in SMs (Psychogios & Tsivgoulis, 2021). This comfort is probably a result of the overwhelming evidence on the benefit from early treatment (Advani et al., 2017; Ajmi et al., 2019; Liberman et al., 2015; Meretoja et al., 2014) combined with the low bleeding risk in IVT‐treated SMs (Kvistad et al., 2019; Pohl et al., 2021). Probably, this has influenced the increasing IVT rates in SMs also at our center, shaping a culture where IVT treatment of SMs is more accepted than avoiding to give IVT to true stroke patients who could have been treated. Yet, as our study period spans over 6 years, other factors may have contributed to the increase of SM rates: Randomized controlled trials established the safety and efficacy of EVT in patients with large vessel occlusions (Berkhemer et al., 2015; Campbell et al., 2015; Jovin et al., 2015; Saver et al., 2015), MRI‐guided thrombolysis for stroke with unknown time of onset was introduced (Thomalla et al., 2018), and thrombolysis in the extended time window was established (Ma et al., 2019; Nogueira et al., 2018). These developments have surely contributed to change the culture of acute stroke treatment toward less restrictions for giving IVT treatment. Simulation training is a tool helping to adapt in this cultural change. It is important to underline that the learning effect of simulation is optimized if directly connected to health service priorities and patient outcomes (translational simulation) (Nickson et al., 2021).

Introduction of MRI in the initial stroke diagnosis algorithm reduces the proportion of SMs treated, increasing the initial diagnostic specificity (Bhattacharya et al., 2013; Burton et al., 2017). Most departments with both modalities available in the emergency room setting have adopted a stroke imaging algorithm containing both CT and MRI modalities (Hetts & Khangura, 2019). This seems unavoidable as imaging of the ischemic penumbra has become increasingly complex, and advanced imaging protocols have been introduced, not only for the extended time window (Albers et al., 2018; Goyal et al., 2020; Ma et al., 2019; Nogueira et al., 2018). Yet, a small but significant percentage of patients with AIS have initial negative MRI scans, and thus, we have IVT treated some SM patients without DWI lesion on initial MRI (Burton et al., 2017; Edlow et al., 2017).

The increase of SMs treated with IVT at our center was not associated with a significant increase in bleeding complications. During the 4‐year study period, only 2 of 156 IVT‐treated SMs were diagnosed with ICH after IVT, and both were clinically asymptomatic. The rate of ICH in SMs is generally low. In the NOR‐TEST study, none of the IVT‐treated SMs exhibited a sICH (Kvistad et al., 2019). A current review concluded with an overall ICH rate as low as 0.7% (Pohl et al., 2021).

Peripheral vertigo was the most frequent SM thrombolyzed in this cohort and the only SM category with a significant increase after implementation of simulation training. In the literature, peripheral vertigo accounts for 23.2% of SMs (Pohl et al., 2021). Before the introduction of simulation‐based team‐training, peripheral vertigo accounted for 2.5% of our patients, rising to 8.6% afterwards (*p* < .001). Peripheral vertigo seems to be a main challenge for stroke physicians and is frequently misinterpreted as stroke in the initial phase. Patients with acute vertigo account for about 4% of all emergency room visits and 20% of emergency room neurological consultations (Newman‐Toker et al., 2008). Although most of the patients presenting with vertigo suffer from benign disorders, up to 27% can end up with serious diagnoses, and cerebral stroke is the underlying cause in 4%–15% of these patients (Newman‐Toker et al., 2008; Royl et al., 2011). The clinical differentiation between peripheral and central causes of vertigo is posing a challenge in the acute setting. Usually, vertigo of cerebrovascular causes is accompanied by other neurological symptoms. Yet, stroke in the posterior circulation can mimic peripheral vestibular syndromes, especially infarction in the territory of the anterior inferior cerebellar artery can present clinically with neuro‐otological symptoms as combined loss of auditory and vestibular functions (Lee et al., 2009).

In order to support clinical decision‐making, the three‐step bedside oculomotor examination HiNTS (Head impulse, Nystagmus, Test of Skew) is a useful tool discriminating central and peripheral causes of vertigo (Kattah et al., 2009). The study emphasizes that the HiNTS examination is more sensitive for stroke than early MRI, the latter potentially being false negative in up to 12% of stroke patients presenting with acute vestibular syndrome during the first 48 h. Systematic testing with HiNTS of patients presenting with dizziness and vertigo has not been part of our clinical routine. We plan to add HiNTS for this patient group with implementation in the clinical routine through simulation training.

The proportion of IVT‐treated SM patients in the other SM categories in our study did not change significantly after the introduction of simulation‐based training. Migraine was the second most common SM, followed by psychiatric disorders and epilepsy (Table 4). Migraine accounts for about 8% of SMs (Pohl et al., 2021). Accompanying aura symptoms can easily be interpreted as a possible stroke, especially if present before or without headache (Otlivanchik & Liberman, 2019). Basilar migraine can present with symptoms from the posterior cerebral artery territory, hemiplegic migraine is rare but poses an obvious challenge in the initial assessment of the patients (Pohl et al., 2021). Psychiatric disorders are responsible for about 10% of all SMs (Pohl et al., 2021). Symptoms are often atypical and fluctuating. Previous psychiatric history is common, and physical examination often reveals inconsistent findings with repeated examinations. The prognosis for full recovery is rather poor with more than one third of patients reporting the same or worse deficits during follow‐up (Gelauff et al., 2014). Seizures are responsible for 13% of SMs (Pohl et al., 2021). Postictal paresis and dysphasia can be misinterpreted as an acute stroke, especially if the seizure was unwitnessed. Early seizures occur in 3.8% of all ischemic stroke cases and 1.5% present with a seizure at the onset of stroke symptoms (Feher et al., 2020). Multimodal brain imaging and the use of MRI in the acute phase may aid in the differentiation of ischemia versus seizure.

Although IVT in SMs is considered to be safe and the cost of undertreatment in acute stroke is high, the high number of IVT‐treated SMs poses a potential medical risk (i.e., ICH) and an economical challenge. Administration of IVT to patients with SM is associated with excess costs, including costs of unnecessary hospital admission, drug‐cost, and the excess cost of a higher care level. The total cost of IVT‐treated SMs in the US (based on 2013 financial estimates) was approximately 15 million dollars per year with an average hospital direct cost of $3600 per admission (Goyal et al., 2015). Understanding which patients constitute the biggest challenge and implementing adequate measures might therefore have important implications. Some patients present with suspected AIS, with symptoms resolving after IVT and with no DWI lesion on follow‐up MRI. MRI can also be false negative in patients with AIS, especially in the posterior fossa (Edlow et al., 2017). This might cause a diagnostic challenge, with some patients actually having suffered from an ischemic event being diagnosed as SM, contributing to some overestimation of SM rates (Edlow et al., 2017). This emphasizes the critical importance of taking a complete medical history and doing a thorough clinical evaluation in the work‐up of patients with suspected SM. An incorrect SM diagnosis may have serious implications for the patients (acute treatment and secondary prophylactic therapy).

This study has several limitations. It is a single center analysis and although we have analyzed over several years, the numbers presented are rather small. Additionally, patients treated with IVT after introduction of simulation‐based team‐training had significantly less atrial fibrillation, possibly lowering the number of true strokes in our study group. Yet, we are presenting consecutive patient treatment result data spanning over several years in the only stroke‐treating hospital in the study's geographical area. Thus, we are able to present population‐based numbers and avoid selection and patient transfer bias. However, a challenge in simulation training in general is to firmly establish a cause‐effect relationship. We found a significant increase in the proportion of SMs among patients who received IVT for presumed AIS after implementation of simulation training. However, as previously discussed, other factors could also have contributed to this increase.

In summary, implementation of in situ simulation‐based team‐training for the acute stroke treatment team seems to be safe, but was associated with a significant increase in the proportion of patients treated with IVT who were later diagnosed as SMs, constituted mainly of patients with peripheral vertigo. This emphasizes the need of accompanying and correcting quality improvement measures tailored to patients presenting with dizziness and vertigo.

## CONFLICT OF INTEREST

The authors declare no conflict of interest.

### PEER REVIEW

The peer review history for this article is available at https://publons.com/publon/10.1002/brb3.2814

## Data Availability

The data that support the findings of this study are available from the corresponding author upon reasonable request.
